# Supplementary material for: Pyruvate Dehydrogenase Kinase 1 inhibition mediated oxidative phosphorylation enhancement in cartilage promotes osteoarthritis progression
Source: BMC Musculoskelet Disord. 2023 Jul 20;24:597. doi: 10.1186/s12891-023-06585-6 (PMC10357736; doi:10.1186/s12891-023-06585-6)
Supplement: Supplementary file 1 — Additional file 1. [file 12891_2023_6585_MOESM1_ESM.zip › KEGG.Gsea.1653622432464/KEGG_REGULATION_OF_AUTOPHAGY.html]

Details for gene set KEGG\_REGULATION\_OF\_AUTOPHAGY[GSEA]

|  || Dataset | 55457\_collapsed\_to\_symbols.cls.cls #OA\_versus\_normal.cls.cls #OA\_versus\_normal\_repos |
| Phenotype | cls.cls#OA\_versus\_normal\_repos |
| Upregulated in class | OA |
| GeneSet | KEGG\_REGULATION\_OF\_AUTOPHAGY |
| Enrichment Score (ES) | 0.19516917 |
| Normalized Enrichment Score (NES) | 0.6713628 |
| Nominal p-value | 0.93028843 |
| FDR q-value | 1.0 |
| FWER p-Value | 1.0 |
Table: GSEA Results Summary

  

Fig 1: Enrichment plot: KEGG\_REGULATION\_OF\_AUTOPHAGY      
 Profile of the Running ES Score & Positions of GeneSet Members on the Rank Ordered List

  

| SYMBOL | TITLE | RANK IN GENE LIST | RANK METRIC SCORE | RUNNING ES | CORE ENRICHMENT || 1 | GABARAPL2 | GABA type A receptor associated protein like 2 [Source:HGNC Symbol;Acc:HGNC:13291] | 776 | 0.575 | 0.0286 | Yes |
| 2 | ATG5 | autophagy related 5 [Source:HGNC Symbol;Acc:HGNC:589] | 1142 | 0.488 | 0.0756 | Yes |
| 3 | IFNA8 | interferon alpha 8 [Source:HGNC Symbol;Acc:HGNC:5429] | 1206 | 0.475 | 0.1441 | Yes |
| 4 | PIK3R4 | phosphoinositide-3-kinase regulatory subunit 4 [Source:HGNC Symbol;Acc:HGNC:8982] | 1424 | 0.439 | 0.1952 | Yes |
| 5 | ULK2 | unc-51 like autophagy activating kinase 2 [Source:HGNC Symbol;Acc:HGNC:13480] | 2796 | 0.270 | 0.1304 | No |
| 6 | ATG4A | autophagy related 4A cysteine peptidase [Source:HGNC Symbol;Acc:HGNC:16489] | 3052 | 0.245 | 0.1485 | No |
| 7 | INS | insulin [Source:HGNC Symbol;Acc:HGNC:6081] | 3885 | 0.166 | 0.1095 | No |
| 8 | IFNA4 | interferon alpha 4 [Source:HGNC Symbol;Acc:HGNC:5425] | 4282 | 0.129 | 0.0987 | No |
| 9 | ATG3 | autophagy related 3 [Source:HGNC Symbol;Acc:HGNC:20962] | 4414 | 0.120 | 0.1071 | No |
| 10 | ATG7 | autophagy related 7 [Source:HGNC Symbol;Acc:HGNC:16935] | 5488 | 0.033 | 0.0289 | No |
| 11 | GABARAP | GABA type A receptor-associated protein [Source:HGNC Symbol;Acc:HGNC:4067] | 5707 | 0.015 | 0.0143 | No |
| 12 | IFNA2 | interferon alpha 2 [Source:HGNC Symbol;Acc:HGNC:5423] | 5869 | 0.004 | 0.0024 | No |
| 13 | IFNA21 | interferon alpha 21 [Source:HGNC Symbol;Acc:HGNC:5424] | 6127 | -0.015 | -0.0153 | No |
| 14 | IFNG | interferon gamma [Source:HGNC Symbol;Acc:HGNC:5438] | 6410 | -0.036 | -0.0317 | No |
| 15 | IFNA10 | interferon alpha 10 [Source:HGNC Symbol;Acc:HGNC:5418] | 6682 | -0.054 | -0.0443 | No |
| 16 | IFNA5 | interferon alpha 5 [Source:HGNC Symbol;Acc:HGNC:5426] | 6755 | -0.059 | -0.0407 | No |
| 17 | IFNA6 | interferon alpha 6 [Source:HGNC Symbol;Acc:HGNC:5427] | 7272 | -0.101 | -0.0653 | No |
| 18 | PIK3C3 | phosphatidylinositol 3-kinase catalytic subunit type 3 [Source:HGNC Symbol;Acc:HGNC:8974] | 8141 | -0.166 | -0.1070 | No |
| 19 | IFNA7 | interferon alpha 7 [Source:HGNC Symbol;Acc:HGNC:5428] | 8533 | -0.196 | -0.1071 | No |
| 20 | IFNA1 | interferon alpha 1 [Source:HGNC Symbol;Acc:HGNC:5417] | 8640 | -0.204 | -0.0839 | No |
| 21 | BECN1 | beclin 1 [Source:HGNC Symbol;Acc:HGNC:1034] | 8644 | -0.204 | -0.0526 | No |
| 22 | PRKAA2 | protein kinase AMP-activated catalytic subunit alpha 2 [Source:HGNC Symbol;Acc:HGNC:9377] | 8864 | -0.222 | -0.0353 | No |
| 23 | ATG12 | autophagy related 12 [Source:HGNC Symbol;Acc:HGNC:588] | 9776 | -0.299 | -0.0599 | No |
| 24 | ULK1 | unc-51 like autophagy activating kinase 1 [Source:HGNC Symbol;Acc:HGNC:12558] | 9798 | -0.301 | -0.0150 | No |
| 25 | ATG4B | autophagy related 4B cysteine peptidase [Source:HGNC Symbol;Acc:HGNC:20790] | 10243 | -0.346 | 0.0039 | No |
| 26 | PRKAA1 | protein kinase AMP-activated catalytic subunit alpha 1 [Source:HGNC Symbol;Acc:HGNC:9376] | 11499 | -0.501 | -0.0161 | No |
| 27 | GABARAPL1 | GABA type A receptor associated protein like 1 [Source:HGNC Symbol;Acc:HGNC:4068] | 12616 | -0.808 | 0.0221 | No |
Table: GSEA details [plain text format]

  

Fig 2: KEGG\_REGULATION\_OF\_AUTOPHAGY      
 Blue-Pink O' Gram in the Space of the Analyzed GeneSet

  

Fig 3: KEGG\_REGULATION\_OF\_AUTOPHAGY: Random ES distribution      
 Gene set null distribution of ES for **KEGG\_REGULATION\_OF\_AUTOPHAGY**
